# Sound Practice and Sound Philosophy

**Published:** 1872-10

**Authors:** 


"*Sound Practice and Sound Philosophy,*" is the title of an essay read by Dr. M. S. Dean, before the Illinois State Dental Society, the following extract of which we take from the proceedings published in the *Missouri Dental Journal:*

"Our practice to be successful, must be based on sound philosophy, that is, upon the knowledge of those principles and laws that govern the materials or agents *with* which we operate, and also those *upon* which we operate. * * * In the filling of very frangible teeth when there is danger to be apprehended—as checking the enamel, or splitting the bicuspids—it was more philosophical to use the heavy leaden mallet, than light steel or wooden ones, as the latter, has greater *vis viva* in proportion to their momentum. When the tooth is very brittle, cuniform pluggers should not be used, as the tapering points acted as wedges and might either check the enamel or split the tooth. The same result may be effected by driving the gold too forcibly into cavities that diminished from their orifices. This evil may result from the use of the heavy lead mallet, but the light mallet would be more cabale of producing this result. It is not sound practice to draw the gold over the margins of a cavity with mallet or burnisher. * * * Gold should not be forced *unwillingly* into any portion of a filling where cohesion is important, nor against the walls of a cavity. The pellet and plug must unite by "first intention" and before the gold has been rolled from place to place and thus become partially condensed or knotted, large pellets should be packed with correspondingly large points and afterwards *condensed* with sharper ones. * * *

"It is sufficient if the walls of the cavity are absolutely protected and the gold sufficiently consolidated to resist the force of mastication. It is more philosophical not to compact very *large cavities* perfectly. In *some respects* a porous filling is better than a perfectly dense one. All gold fillings are porous to some extent, and there is little danger of getting them too compact. * * * It cannot be possible to pack gold more accurately against *concave* walls with instruments having a smooth, *flat* surface, than with a serrated *convex* one. If the surface of the filling could be kept flat, no points could be better adapted to the purpose of making dense fillings, but this can rarely be done, and when it is not, no points are better adapted to shingle or bridge over the interstices and deceive the operator. It has recently been asserted by a writer who uses the "steel mallet and smooth points," that with these, operations are performed, giving no pain to the patient, and with no risk of fracturing the enamel, and that the fillings are harder and better adapted to the margins of the cavity. Whether this magic power resides mostly in the steel mallet or smooth points, or whether they are equally efficacious in producing these *desirable* results, and in averting the contrary ones, the writer has neglected to state. Most dentists place a layer, or cushion as it were, of gold between the instrument and the enamel. If this is done these points would as readily fracture the enamel as if they were finely serrated. If this inference is correct we are forced to conclude that this charm belongs to the peculiar properties of the steel of which this wonderful mallet is made, and we are warranted in this conclusion by the assurance of the writer that it is *non vibrating*. * * * By beating the gold under a smooth pointed plugger, it is drawn snugly and perfectly against the walls and margins of the cavity, on the same principle as a cubic slug is made to adjust itself to the side of a gun barrel when vigorously rammed down with a steel ramrod. If the enamel were made of steel this would be sound practice, provided our patients were made of steel also. * * *

"Success in the treatment of alveolar abscess depends in a good measure upon sound philosophy. One or two thorough applications of creosote or carbolic acid are usually sufficient. The continued and daily applications afterwards often produce the trouble instead of relieving it. * * Those having a fistulous opening are easily treated and quickly cured, as the irritating particles may be washed out by injections. Hence great care should be exercised, when there is no opening, to remove patiently and thoroughly all extraneous matter from the canals of roots before therapeutic remedies are applied. * * *

"The universal practice of restoring the contour of the teeth is not sound practice, in many cases it was important to mastication and the articulation of the voice, and also to the beauty of these organs, but the practice was becoming too universal. Many cases were instanced where they must fail, and when fillings having an inclining surface so that the force of mastication would be in a direction to retain them, is more philosophical."

---

